# Phenylketonuria in Portugal: Genotype–phenotype correlations using molecular, biochemical, and haplotypic analyses

**DOI:** 10.1002/mgg3.1559

**Published:** 2021-01-19

**Authors:** Filipa Ferreira, Luísa Azevedo, Raquel Neiva, Carmen Sousa, Helena Fonseca, Ana Marcão, Hugo Rocha, Célia Carmona, Sónia Ramos, Anabela Bandeira, Esmeralda Martins, Teresa Campos, Esmeralda Rodrigues, Paula Garcia, Luísa Diogo, Ana Cristina Ferreira, Silvia Sequeira, Francisco Silva, Luísa Rodrigues, Ana Gaspar, Patrícia Janeiro, António Amorim, Laura Vilarinho

**Affiliations:** ^1^ Newborn Screening, Metabolic and Genetics Unit Department of Human Genetics National Institute of Health Dr Ricardo Jorge Porto Portugal; ^2^ i3S – Instituto de Investigação e Inovação em Saúde Universidade do Porto Porto Portugal; ^3^ IPATIMUP – Instituto de Patologia e Imunologia Molecular da Universidade do Porto Porto Portugal; ^4^ FCUP ‐ Faculty of Sciences University of Porto Porto Portugal; ^5^ Inherited Metabolic Disease Reference Center Pediatric Department Centro Hospitalar Universitário do Porto Porto Portugal; ^6^ Metabolic Diseases Unit Pediatric Department University Center São João Hospital ‐ HSJ Porto Portugal; ^7^ Inherited Metabolic Disease Reference Center Pediatric Hospital, Hospital and University Center of Coimbra Coimbra Portugal; ^8^ Metabolic Unit Hospital Dona Estefânia Centro Hospitalar Universitário Lisboa Central Lisbon Portugal; ^9^ Pediatric Department Hospital Central of Funchal Funchal Portugal; ^10^ Pediatrics Department Hospital of Divino Espírito Santo of Ponta Delgada, EPE, Ponta Delgada Azores Portugal; ^11^ Inherited Metabolic Disease Reference Center Lisbon North University Hospital Center (CHULN), EPE Lisboa Portugal; ^12^ Research and Development Unit Department of Human Genetics National Institute of Health Dr Ricardo Jorge Porto Portugal

**Keywords:** biochemical and genetic findings, haplotypic study, mutation spectrum, phenylketonuria, Portuguese population

## Abstract

**Background:**

The impairment of the hepatic enzyme phenylalanine hydroxylase (PAH) causes elevation of phenylalanine levels in blood and other body fluids resulting in the most common inborn error of amino acid metabolism (phenylketonuria). Persistently high levels of phenylalanine lead to irreversible damage to the nervous system. Therefore, early diagnosis of the affected individuals is important, as it can prevent clinical manifestations of the disease.

**Methods:**

In this report, the biochemical and genetic findings performed in 223 patients diagnosed through the Portuguese Neonatal Screening Program (PNSP) are presented.

**Results:**

Overall, the results show that a high overlap exists between different types of variants and phenylalanine levels. Molecular analyses reveal a wide mutational spectrum in our population with a total of 56 previously reported variants, most of them found in compound heterozygosity (74% of the patients). Intragenic polymorphic markers were used to assess the haplotypic structure of mutated chromosomes for the most frequent variants found in homozygosity in our population (p.Ile65Thr, p.Arg158Gln, p.Leu249Phe, p.Arg261Gln, p.Val388Met, and c.1066‐11G>A).

**Conclusion:**

Our data reveal high heterogeneity at the biochemical and molecular levels and are expected to provide a better understanding of the molecular basis of this disease and to provide clues to elucidate genotype–phenotype correlations.

## Graphical abstract

The present study identify and characterize the variants underlying PKU in affected individuals in the Portuguese PKU/HPA cohort. Biochemical data, haplotic analysis and genetic findings performed in 223 patients diagnosed through the Portuguese Neonatal Screening Program (PNSP) is presented. The information obtained will improve the diagnostic applicability of mutational analysis and the capacity to predict the evolution of the disease.

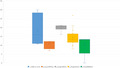

## 1 INTRODUCTION

Phenylketonuria (PKU; OMIM #261600) is an autosomal recessive genetic disorder caused by the deficiency in the hepatic enzyme phenylalanine hydroxylase (PAH; OMIM #612349) (Scriver et al., 1995) that converts phenylalanine into tyrosine requiring the cofactor tetrahydrobiopterin (BH4). Whenever the enzymatic activity of PAH (EC 1.14.16.1) is impaired, the essential amino acid phenylalanine cannot be hydroxylated into tyrosine, resulting in the elevation of phenylalanine, and its metabolic derivatives in blood and other body fluids. The high blood levels of phenylalanine can result in growth failure, microcephaly, seizures, and psychomotor/intellectual deficit and growth failure, due to a marked accumulation of phenylalanine and the toxic products of its metabolism (Erlandsen & Stevens, 1999; Madden, 2004). When phenylalanine in high levels cross the blood–brain barrier, it causes irreversible structural damage to the central nervous system (Anderson & Leuzzi, 2010). The enzyme PAH requires a cofactor; the tetrahydrobiopterin (BH4), which is also the cofactor in tyrosine and tryptophan hydroxylation reactions. About 1%–2% of cases of hyperphenylalaninemia (HPA) are due to variants in genes coding for enzymes involved in BH4 biosynthesis or regeneration (Blau et al., 2001; Thöny & Blau, 2006). However, some patients with defects in BH4 biosynthesis, such as in Segawa disease and sepiapterin reductase deficiency, do not show hyperphenylalaninemia (Bonafé et al., 2001; Ichinose et al., 1994). In addition, certain variants of PKU are responsive to BH4 (Trefz et al., 2009).

The human *PAH* gene covers approximately 100 kb of genomic DNA and encodes a protein of 452 amino acids, which are assembled in a functional homotetramer. This gene consists of 13 exons and 12 introns, and has been mapped on chromosome 12, band region q23.2 (Donlon et al., 2014; Scriver et al., 1995). Over 1000 variants in the *PAH* gene have been associated with PKU in the *PAH*vdb (Phenylalanine Hydroxylase Gene Locus‐Specific Database, *PAH*db; http://www.pahdb.mcgill.ca/), and BIOPKU (http://www.biopku.org) databases, primarily from Caucasian populations (Blau et al., 2014). Exonic point mutations comprise about 90% of all variants and the next most frequently occurring type is the splice junction variant c.1066‐11G>A (IVS10‐11G>A) (Birk et al., 2007; Guldberg, Henriksen, et al., 1993; Pérez et al., 1997). The position and nature of the variant dictate its effect on the activity of the PAH enzyme, which determines the hyperphenylalaninemia phenotype of the patient. Low or nonenzymatic activity results in the classic phenylketonuria phenotype (MIM **#**261600). Other variants only partly inhibit the enzyme activity, resulting in mild phenylketonuria or mild hyperphenylalaninemia. The frequency of the PKU disease and distribution of the *PAH* gene variants differs between populations. In Europe, the prevalence is about 1:10,000 newborns (Loeber, 2007) but in some areas is higher (Blau et al., 2010) as is for instance in the Andalusia population, the incidence of this disorder is about 1:12,000 (Delgado et al., 2011). Persistent hyperphenylalaninemia is detected in about 1:4000 live births in Turkey because of the high consanguinity (Ashraf El‐Metwally et al., 2018) and in Northern Ireland (Ozalp et al., 2001; Zschocke et al., 1997). Finland has the lowest PKU prevalence in Europe with 1:100,000 newborns, and for this reason, this disease is not included in their neonatal screening program (Guldberg et al., 1995; Scriver & Kaufman, 2001). In the United States of America, the prevalence is 1:15,000 (NIH, 2000). In Latin America, it varies from about 1:50,000 to 1:25,000 births being generally higher in southern Latin America (Borrajo, 2007). The prevalence of PKU varies from 1:15,000 to 1:100,500 births in certain regions of China (Jiang et al., 2003; Zhan et al., 2009), but is less than 1:200,000 in Thailand (Pangkanon et al., 2009), and approximately, 1:70,000 in Japan (Aoki et al., 2007). In the Iranian population, the PKU prevalence is higher, due to consanguinity, 1:6250 to 1:3704 (Ghiasvand et al., 2009; Habib et al., 2010; Koochmeshgi et al., 2002; Vallian et al., 2003). Africa seems to have a very low prevalence of phenylketonuria (NIH, 2000).

### 1.1 Brief PKU history

Until the 60 s, most children born with phenylketonuria became neurologically disabled. In 1934, Fölling and Über (1934) identified an excess of phenylketone bodies (a metabolites of phenylalanine) as the cause of a strange, musty odor from the urine of two affected individuals. In 1953, Bickel reported the effectiveness of a low phenylalanine diet in a child with PKU (Bickel et al., 1953). Later, in the 1960s, Robert Guthrie developed a bacterial inhibition test that could detect high amounts of phenylalanine in a single dried blood spot (Guthrie, 1961; Guthrie & Susi, 1963). The “Guthrie test” made possible to carry out newborn screening testing for PKU, enabling early diagnosis and dietary treatment of the disease and prevention of the development of intellectual disability (Wegberg et al., 2017). Nowadays, many countries around the world, including Portugal since 1979, integrate PKU, in their neonatal screening program (Blau et al., 2010).

The present study aims to identify and characterize the variants underlying PKU in affected individuals in the Portuguese PKU/HPA cohort, for a better understanding of this disease. The information obtained will improve the diagnostic applicability of mutational analysis and the capacity to predict the evolution of the disease.

## 2 MATERIALS AND METHODS

### 2.1 Patients

A total of 377 PKU/HPA patients were detected by the Portuguese Newborn Screening Program from 1979 to 2018. Most samples were collected between the third and sixth days of life.

The cohort here studied represents approximately 58% (223/377) of the patients followed at the clinical reference centers. The remaining 154 patients have molecular study made in another metabolic laboratory center (Rivera et al., 2011). Most of the patients were diagnosed by the newborn screening program. The diagnosis of PKU/HPA is suspected when a blood phenylalanine level ≥2.45 mg/dL (148 μmol/L) in a newborn screening sample is found. Newborns with blood phenylalanine levels persistently ≥2.45 mg/dL and a Phe/Tyr ratio >1.5 are referred to treatment centers. Dietary treatment (phenylalanine restriction) is implemented if levels are ≥5.94 mg/dL (360 μmol/L).

### 2.2 Molecular genetic analysis

Genomic DNA was automatically extracted from whole blood or dried blood spots, using an automated method (EZ1 DNA Blood 350 μl, or EZ1 DNA tissue kit, QIAGEN). The 13 protein‐coding exons and flanking intronic sequences of *PAH* gene (GenBank sequence: NM_000277.3; ENSG00000171759; ENST00000553106.6) were directly sequenced after PCR amplification in an ABI PRISM™ 3130XL Genetic Analyzer (Applied Biosystems, Foster City, CA, USA). Primers for the 13 exons and exonic/intronic boundaries of the *PAH* gene were designed employing the NCBI Primer‐BLAST tool (http://www.ncbi.nlm.nih.gov/tools/primer‐blast/) (see Table S1). These primers were tagged with a M13 sequence for the later cycle sequencing reaction. PCR was carried out using the EmeraldAmp MAX PCR Master Mix (Takara Bio Inc., Kusatsu, Shiga, Japan). PCR products were purified with ExoSAP‐IT (Affymetrix, Santa Clara, CA, USA), and subjected to a cycle sequencing reaction using BigDye Terminators v3.1 kit (Applied Biosystems, Foster City, CA, USA), and M13 primers [M13(‐21)F: 50‐TGTAAAACGACGGCCAGT‐30, M13R: 50‐CAGGAAACAGCTATGACC‐30].

Protein sequences were aligned in Geneious v5.4 using the default options (Drummond et al., 2010). The observed variants were referred to the NCBI reference sequence for human *PAH* gDNA (NG_008690.2.)

### 2.3 Haplotypic analyses of the most frequent variants

We used the information obtained from six previously described polymorphic single nucleotide polymorphisms (SNPs) [p.Gln232= (rs1126758), p.Val245= (rs1042503), p.Leu385= (rs772897), c.168+19T>C (IVS2+19T>C; rs17842947), c.441+47C>T (IVS4+47C>T; rs1718301) e c.510‐54G>A (IVS5‐54G>A; rs2251905)] in order to establish the haplotypic background of homozygous patients for the six most frequent PAH disease‐associated variants.

### 2.4 Editorial policies and ethical considerations

This study was approved by the institutional review board of the Ethics Committee of National Health Institute Dr. Ricardo Jorge on 03rd June 2020. All procedures were followed in accordance with the ethical standards of the responsible committee on human experimentation (institutional and national) and with the Helsinki Declaration of 1975, as revised in 2000 and approved by the Ethics Committees.

## 3 RESULTS

### 3.1 Mutational spectrum

The *PAH* molecular analysis of the 223 patients revealed 56 distinct variants distributed in 129 genotype combinations (Tables 1 and S1). Most patients (73.5% of the cohort) were heterozygous compounds. Among the homozygous individuals 17 carry the c.782G>A (p.Arg261Gln) variant, 10 carry the c.1066‐11G>A (IVS10‐11G>A) variant, 6 carry the c. 473G>A (p.Arg158Gln), and 5 patients the c.1162G>A (p.Val388Met) variant. The remaining patients harbor one of the following variants: c.194T>C (p.Ile65Thr), c.204A>T (p.Arg68Ser), c.385G>T (p.Asp129Tyr), c.526C>T (p.Arg176*), c.727C>T (p.Arg243*), c.745C>T (p.Leu249Phe), c.754C>T (p.Arg252Trp), c.809G>A (p.Arg270Lys), c.842C>T (p.Pro281Leu), c.1229T>G (p.Phe410Cys), c.168+5G>A (IVS2+5G>A), and c.441+5G>T (IVS4+5G>T). Concerning the type of variant found, the majority are missense replacements (76.8%), followed by variants at splice sites (12.5%), nonsense (5.4%), and frameshift deletions (5.4%). The two most prevalent pathogenic variants in our population are the c.782G>A (p.Arg261Gln) found in 63 instances (14.13%) and the splicing variant c.1066‐11G>A (IVS10‐11G>A), found in 60 (13.45%) followed by the replacements c.1162G>A (p.Val388Met) and c.194T>C (p.Ile65Thr), 37 times each (8.3%).

**TABLE 1:** Mutational spectrum of PKU Portuguese patients

PAH mutation	No alleles	Allele frequency (%)	DNA change	Type	Gene region	Protein domain	PAH activity (%)
p.(Phe39Leu)	1	0.22	c.117C>G	Missense	Exon 2	Regulatory	49
p.(Gly46Ser)	2	0.45	c.136G>A	Missense	Exon 2	Regulatory	16
p.(Leu48Ser)	1	0.22	c.143T>C	Missense	Exon 2	Regulatory	39
p.(Arg53His)	1	0.22	c.158G>A	Missense	Exon 2	Regulatory	79
p.(Phe55Leu)	1	0.22	c.165T>G	Missense	Exon 2	Regulatory	na
p.(Ile65Thr)	37	8.30	c.194T>C	Missense	Exon 3	Regulatory	33
p.(Arg68Ser)	7	1.57	c.204A>T	Missense	Exon 3	Regulatory	68
p.(Ser87Arg)	1	0.22	c.261C>A	Missense	Exon 3	Regulatory	24
p.(Asp129Gly)	2	0.45	c.386A>G	Missense	Exon 4	Regulatory	na
p.(Asp129Tyr)	10	2.24	c.385G>T	Missense	Exon 4	Catalytic	na
p.(Asp145Val)	2	0.45	c.434A>T	Missense	Exon 4	Catalytic	na
p.(Arg158Gln)	24	5.38	c.473G>A	Missense	Exon 5	Catalytic	10
p.(Ile164Val)	2	0.45	c.490A>G	Missense	Exon 5	Catalytic	na
p.(Arg176Leu)	19	4.26	c.527G>T	Missense	Exon 6	Catalytic	42
p.(Arg176*)	11	2.47	c.526C>T	Nonsense	Exon 6	Catalytic	<1
p.(Glu178Gly)	1	0.22	c.533A>G	Missense	Exon 6	Catalytic	39
p.(Glu182Lys)	1	0.22	c.544G>A	Missense	Exon 6	Catalytic	na
p.(Val230Ile)	1	0.22	c.688G>A	Missense	Exão 6	Catalytic	63
p.(Arg241His)	1	0.22	c.722G>A	Missense	Exão 7	Catalytic	23
p.(Arg243Gln)	6	1.35	c.728G>A	Missense	Exão 7	Catalytic	14
p.(Arg243*)	2	0.45	c.727C>T	Nonsense	Exão 7	Catalytic	<1
p.(Leu249Phe)	22	4.93	c.745C>T	Missense	Exão 7	Catalytic	na
p.(Arg252Trp)	18	4.04	c.754 C>T	Missense	Exon 7	Catalytic	<1
p.(Arg261Gln)	63	14.13	c.782 G>A	Missense	Exon 7	Catalytic	44
p.(Arg261*)	1	0.22	c.781C>T	Nonsense	Exon 7	Catalytic	1
p.(Arg270Lys)	10	2.24	c.809G>A	Missense	Exon 7	Catalytic	<1
p.(Pro281Leu)	15	3.36	c.842C>T	Missense	Exon 7	Catalytic	2
p.(Arg297Cys)	5	1.12	c.889C>T	Missense	Exon 8	Catalytic	na
p.(Arg297His)	1	0.22	c.890G>A	Missense	Exon 8	Catalytic	21
p.(Ala300Ser)	8	1.79	c.898G>T	Missense	Exon 8	Catalytic	31
p.(Leu308Phe)	1	0.22	c.922C>T	Missense	Exon 9	Catalytic	na
p.(Ala309Asp)	1	0.22	c.926C>A	Missense	Exon 9	Catalytic	na
p.(Ala309Val)	2	0.45	c.926C>T	Missense	Exon 9	Catalytic	42
p.(Ala313Val)	1	0.22	c.938C>T	Missense	Exon 9	Catalytic	na
p.(Ala322Gly)	1	0.22	c.965C>G	Missense	Exon 9	Catalytic	75
p.(Leu348Val)	11	2.47	c.1042C>G	Missense	Exon 10	Catalytic	35
p.(Ser359Leu)	1	0.22	c.1076 C>T	Missense	Exon 11	Catalytic	na
p.(Leu367Gln)	1	0.22	c.1100T>A	Missense	Exon 11	Catalytic	na
p.(Val388Met)	37	8.30	c.1162G>A	Missense	Exon 11	Catalytic	28
p.(Glu390Gly)	5	1.12	c.1169A>G	Missense	Exon 11	Catalytic	62
p.(Ala403Val)	11	2.47	c.1208C>T	Missense	Exon 12	Catalytic	66
p.(Arg408Trp)	2	0.45	c.1222C>T	Missense	Exon 12	Catalytic	2
p.(Phe410Cys)	3	0.67	c.1229T>G	Missense	Exon 12	Oligomerization	na
p.(Tyr414Cys)	4	0.90	c.1241A>G	Missense	Exon 12	Oligomerization	57
p.(Asp415Asn)	4	0.90	c.1243G>A	Missense	Exon 12	Oligomerization	72
p.(Ile421Thr)	1	0.22	c.1262T>C	Missense	Exon 12	Oligomerization	na
IVS2+5G>A	8	1.79	c.168+5G>A	Splicing	Intron 2	_	na
IVS2+5G>C	1	0.22	c.168+5G>C	Splicing	Intron 2	_	na
IVS4+5G>T	2	0.45	c.441+5G>T	Splicing	Intron 4	_	na
IVS7+1G>A	1	0.22	c.842+1G>A	Splicing	Intron 7	_	na
IVS10‐11G>A	60	13.45	c.1066‐1 1G>A	Splicing	Intron 10	_	5
IVS11+5G>A	1	0.22	c.1199+5G>A	Splicing	Intron 11	_	na
IVS12+1G>A	5	1.12	c.1315+1G>A	Splicing	Intron 12	_	<1
p.(Phe55Leufs*6)	4	0.90	c.165delT	Frameshift deletion	Exon 2	Regulatory	na
p.(Gly352Valfs*12)	1	0.22	c.1055delG	Frameshift deletion	Exon 10	Catalytic	na
p.(Gly352Valfs*48)	1	0.22	c.1055delG	Frameshift deletion	Exon 10	Catalytic	na

Note

The in vitro relative residual activity of PAH according to the *PAH*db (http://www.pahdb.mcgill.ca) is also indicated (na – not available) (GenBank: NM_000277.3; ENSG00000171759; ENST00000553106.6).

These causative variants are distributed as follows: 35 in the catalytic domain (62.5%), 10 in the regulatory domain (17.85%), 4 in the tetramerization domain (7.14%), and 7 in the intronic regions (12.5%). No causative variants were found in exon 1 and 13. The density of variants is higher in exon 9, which presents five variants dispersed by only 54 nucleotides. Previous studies have shown a higher frequency of *PAH* variants in exon 7 (Abadie et al., 1989; Dworniczak, Kalaydjieva, et al., 1991; Hamzehloei et al., 2012; Vallian et al., 2003; Zare‐Karizi et al., 2011). In fact, in our cohort, the number of variants found in exon 7 is higher than the ones found in other exons, but when the exon size is taken into account the highest proportion of variants is observed in exon 9.

### 3.2 Biochemical phenotypic spectrum

**FIGURE 1:**
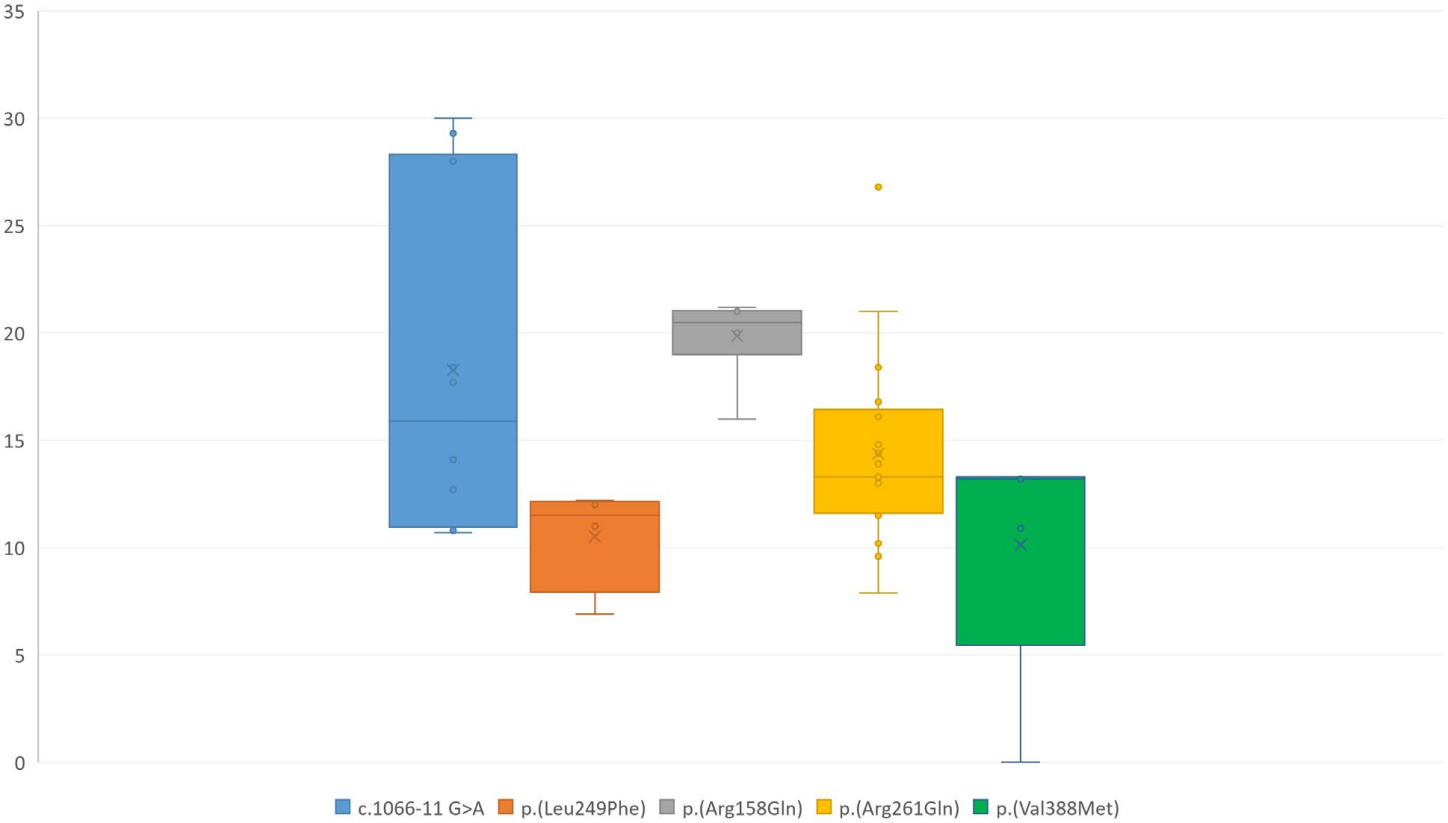
Levels of phenylalanine mutations found in the most frequent homozygous individuals (at the neonatal screening)

Plasmatic blood levels of phenylalanine at the neonatal screening are shown in Table S1. The relationship between the biochemical phenotype (phenylalanine levels) and the molecular genotype in homozygous patients for five variants is shown in Figure 1. The variant associated with the wider spectrum of phenylalanine levels is the c.1066‐11G>A, for which values ranging from 10.7 to 30 mg/dL are present in homozygous patients. Individuals homozygous for the c.745C>T (p.Leu249Phe) shows the lowest levels of phenylalanine (average 10.53 mg/dL). The most common variant c.782G>A (p.Arg261Gln) is associated with a mean value of 14.37 mg/dL, whereas the c.473G>A (p.Arg158Gln) and c.1162G>A (p.Val388Met) variants reach values of 19.86 mg/dL and 12.69 mg/dL, respectively.

### 3.3 Haplotypic analyses of the most frequent variants

**FIGURE 2:**
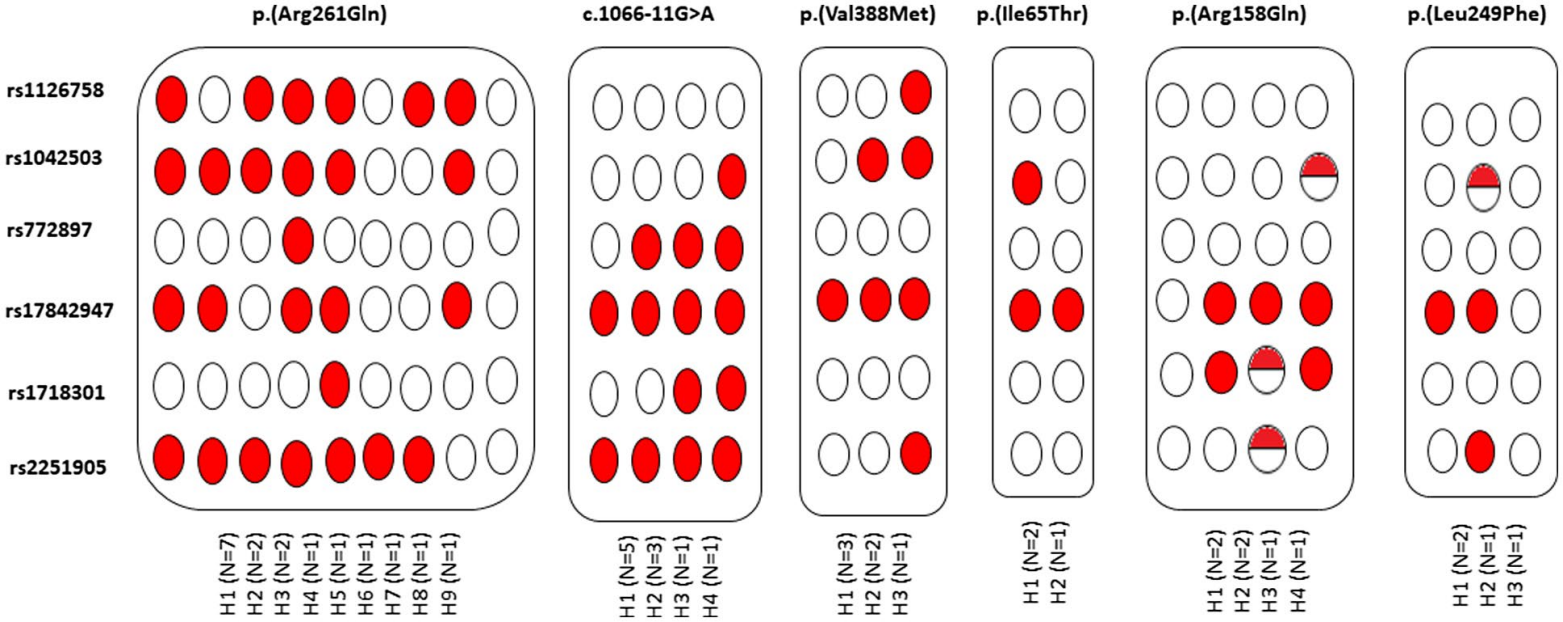
Haplotypes observed in homozygous individuals carrying the most frequent variants in this study defined by six polymorphic single nucleotide polymorphisms (SNPs) [p.Gln232= (rs1126758), p.Val245= (rs1042503), p.Leu385= (rs772897), c.168+19T>C (IVS2+19T>C; rs17842947), c.441+47C>T (IVS4+47C>T; rs1718301) e c.510‐54G>A (IVS5‐54G>A; rs2251905)]. (Individuals carrying the ancestral alleles are shown in unfilled circles, homozygosity for the derivate alleles is shown in red circles and heterozygosity is shown in half filled circles) (GenBank: NM_000277.3; ENSG00000171759; ENST00000553106.6)

The information obtained with six previously described polymorphic single nucleotide polymorphisms (SNPs) was used to establish the haplotypic background of homozygous patients for the six most frequent disease‐associated *PAH* variants (Figure 2). For the c.782G>A (p.Arg261Gln) variant, seven patients carry the most frequent haplotype (H1). Four patients carry H2 and H3 and both allelic combinations differ only in a single position in relation to H1, from which they may have been derived.

The remaining six patients show six distinct haplotypes indicating a high level of diversity, which is in accordance to the fact that c.782G>A (p.Arg261Gln), locates at a hypermutable CpG dinucleotide and these may well represent independent origins for this variant (Zschocke & Hoffmann, 1999). For the c.1066‐11G>A, five out of ten homozygous patients revealed the same haplotype (H1) and other three patients carry the haplotype H2, which differs from H1 in only the rs17842947‐C allele. The variant c.1162G>A (p.Val388Met) show three distinct haplotypes yet H1 and H2 differ by one allele at the marker rs171830. H3 differs from H2 at two positions; since this variant is not in a CpG site, it is likely that H2 and H3 haplotypes derivate from the ancestral H1.

The c.473G>A (p.Arg158Gln) variant shows two more frequent haplotypes that differ at two positions, it is likely that it may have occurred at least two independent times. The variant c. 194T>C (p.Ile65Thr) shows two haplotypes with a single allelic difference between them suggesting a single origin.

The c.745C>T (p.Leu249Phe) reveals a pattern that is consistent with a single origin but only if there has been a back mutation regarding the polymorphism rs17842947. If this was not the case, it is possible that two independent events will have contributed to the birth of this variant.

## 4 DISCUSSION

In the late 70 s, the Portuguese Neonatal Screening Program was established by the Ministry of Health, with phenylketonuria (PKU) and later, in 1981, with congenital hypothyroidism (CH) screening (Magalhães et al., 1986; Magalhães & Osório, 1984; Osório et al., 1992; Osório & Soares, 1987). PNSP is performed in the whole Country and centralized in a single laboratory of the National Health Institute Dr. Ricardo Jorge, in Porto, that receives over 400 samples per day (86,000/year). Until 2004, the phenylalanine value was obtained using the Quantase™ neonatal phenylalanine screening kit. In 2004, the development of electrospray tandem mass spectrometry (MS/MS) allowed the use of a single test to screen for 24 treatable inherited inborn errors of metabolism, plus congenital hypothyroidism, and since 2013, also cystic fibrosis (Marcão et al., 2018; Sousa et al., 2015; Vilarinho et al., 2010).

Currently, PNSP is the state reference for the newborn screening, diagnosis, and follow‐up of patients with PKU and is considered a medical success story. In Portugal, approximately 377 HPA patients are followed, and from these, 345 are PKU. The birth prevalence of PKU in Portugal is estimated to be 1:10,772 (Newborn Screening Program – Annual Report 2018).

In our cohort, the most prevalent variant is c.782G>A (p.Arg261Gln) variant (14.13%, Table 1). Its frequency, in our study, is similar to that found in south European and Mediterranean countries (Zare‐Karizi et al., 2011). The haplotypic analysis revealed a predominant combination in the Portuguese patients although independent origins can also be hypothesized given the fact that involves a CpG site.

The second most prevalent variant is the intronic c.1066‐11G>A (IVS10‐11G>A) variant (13.45%). This variant is also the second most common variant in the *PAH* knowledgebase. Given its high frequency in the Mediterranean area, c.1066‐11G>A has been considered the “Mediterranean variant” (Aldámiz‐Echevarría et al., 2016; Okano et al., 1991; Rivera et al., 2011; Vieira Neto et al., 2018; Zschocke, 2003). Interestingly, when we look to its world distribution, it seems to have a decreasing rate from the east to the west of the Mediterranean areas trough range expansion probably during the Neolithic period with the highest relative frequencies in Turkey (32%) (Ozgüç et al., 1993), Iran (26.07%) (Bonyadi et al., 2010; Hamzehloei et al., 2012; Zare‐Karizi et al., 2011), Bulgaria (25%) (Berthelon et al., 1991), Greece (12.5%) (Traeger‐Synodinos et al., 1994), south Italy (8.8%) (Daniele et al., 2009; Dianzani et al., 1995; Giannattasio et al., 2001), and Spain (9.7%) (Aldámiz‐Echevarría et al., 2016; Bueno et al., 2013; Desviat et al., 1999). In this context, the term “Mediterranean variant” for c.1066‐11 G>A has been suggested to be expanded to “Southern Eurasian variant” (Kostandyan et al., 2011). Accordingly, this variant could have had a Turkish origin and subsequent spread throughout the Mediterranean countries (Scriver & Kaufman, 2001). Haplotypic data (Figure 2) are in accordance with previous studies (Rivera et al., 1997, 2011; Vieira Neto et al., 2018; Zschocke, 2003; Zschocke & Hoffmann, 1999) that claim a single origin for this allele.

The third most common variant found in this study is the c.194T>C (p.Ile65Thr) variant. It is also quite common in Spain and Ireland (Zschocke, 2003). It has been suggested that this variant originated during the Paleolithic in Western Europe (Zschocke, 2003) and haplotypic data are indicative of a single origin although conclusive interpretations are difficult given the low number of homozygous individuals analyzed in this study (*N* = 3).

Apart from c.782G>A (p.Arg261Gln) and c.1066‐11G>A variants, other variants were found with also a considerably high frequency in our study population: c.473G>A (p.Arg158Gln), c.527G>T (p.Arg176Leu), c.745C>T (p.Leu249Phe), c.754C>T (p.Arg252Trp), c.842C>T (p.Pro281Leu); c.526C>T (p.Arg176*), c.1042C>G (p.Leu348Val), c.1208C>T (p.Ala403Val), c.385G>T (p.Asp129Tyr) and c.809G>A (p.Arg270Lys) (5.38–2.24%). Also noteworthy is the finding that only one individual (Table 1) in our population carries the c.1222C>T (p.Arg408Trp) variant in heterozygosity, the most prevalent PKU causing variant reported to date in the world (Zschocke, 2003). This variant is the major PKU causing variant in northern Europe (Giannattasio et al., 1997; Lilleväli et al., 1996, 2019), arising from at least two independent events in Eastern Europe and the British Isles (Aulehla‐Scholz & Heilbronner, 2003; Dworniczak, Aulehla‐Scholz, et al., 1991, 1991).

Another interesting fact is that all variants identified in our population have already been described in other populations apart from c.809G>A (p.Arg270Lys), which frequency in our study is 2.24%. As reported by Rivera et al. (2011), this variant has only been identified in patients with Portuguese ancestry (Vieira Neto et al., 2018), which indicates a local origin.

Herein, we report the mutational spectrum of PAH deficiency in a cohort of 223 patients in the Portuguese population studied over 40 years (1980–2018). We observed a high level of genetic heterogeneity with 56 different variants distributed to 129 different genotypes, most of them falling into the category of missense type (76.8%). These results are in accordance with previous reports from other south European and Mediterranean studies, but are clearly different from those results found in North and Central European countries where the prevalent variants are c.1315+1G>C (IVS12+1G>C) and c.1222C>T (p.Arg408Trp) (Guldeberg et al., 1998; Guldberg, Henriksen, et al., 1993; Gundorova et al., 2019; Jaruzelska et al., 1993). In this study, the four most prevalent variants are c.782G>A (p.Arg261Gln), c.1066‐11G>A, c.1162G>A (p.Val388Met) and c.194T>C (p.Ile65Thr), representing 44.17% of the total alleles. These frequencies are in concordance with previous studies involving the Portuguese population (Acosta et al., 2001; Osório et al., 1992;; Vieira Neto et al., 2018; Vilarinho et al., 2011, 2006, 2018).

The results obtained from molecular analyses can be indicative of the degree of protein dysfunction, residual PAH activity and consequently the metabolic phenotype. The classification of *PAH* genotypes allows the prediction of the biochemical and metabolic phenotypes in many genotypes and can be useful for the management of HPA in newborns (Gámez et al., 2000; Kayaalp et al., 1997; Waters, 2003). PKU always causes HPA, but not all HPA are PKU. Children with “atypical” or “malignant” PKU due to a deficiency in the cofactor for PAH; BH4; do not respond to dietary phenylalanine restriction (Blau, 2008, 2016; Blau et al., 2001; Smith et al., 1975). Differential diagnosis of HPA is critical to distinguish infants with PAH deficiency from those with HPA and those who have BH4 deficiency.

Our data on the levels of phenylalanine at the newborn screening in homozygous individuals may provide some useful information in relation to the establishment of the dietary limits for phenylalanine intake. For instance, in relation to the c.1066‐11G>A, a wide range of phenylalanine levels is shown by homozygous individuals (see Figure 1), however, less disperse values were detected for c.473G>A (p.Arg158Gln) homozygous individuals, although both variants are associated with low residual enzymatic activity (5% and 10%, respectively, Table 1).

Nowadays, PKU diagnosis also relies on in vitro expression analysis of recombinant mutant proteins. Several studies revealed that in general severe loss of function variants (such as splicing, nonsense or severe missense variants), which display in vitro null/reduced residual activity, are associated with the most severe forms of the disease (Santos et al., 2006, 2010) (Table 1). Early diagnosis and prompt intervention have undoubtedly allowed most individuals with PKU to avoid severe mental disability. Dietary restriction of phenylalanine remains the mainstay of treatment but PKU is an active area of research and new treatment options are emerging that might reduce the burden of the difficult and restrictive diet on patients and their families (Enacán et al., 2019; Giovannini et al., 2012; Harding & Blau, 2010; Sumaily & Mujamammi, 2017; Walter et al., 2002). On the contrary, patients with gene variants that determine a high residual enzyme activity (those with the mildest metabolic phenotypes) have a higher probability of responding to BH4 (Bueno et al., 2013; Michals‐Matalon et al., 2007; Rivera et al., 2011; Staudigl et al., 2011; Vieira Neto et al., 2019). In this regard, patients with a genotype known to be non‐BH4‐responsive should not undergo BH4 testing, while patients with a genotype with BH4‐responsive variations may directly proceed to a treatment trial rather than a BH4 loading test. In all other patients, a BH4 loading should be considered.

## 5 CONCLUSION

PKU occupies a unique place in the history of metabolic diseases, as not only the most commonly known inborn error of amino acid metabolism to be identified, but also the first genetic inborn metabolic disease to be screened in the neonatal screening program. PKU is also the first serious genetic condition to be treated effectively, allowing affected individuals to lead a fulfilling life (Camp et al., 2014).

Due to the current newborn screening program, treatment can be started shortly after birth and the patients fall within the broad normal range of general ability, attaining expected educational standards and have independent lives as adults. Differential diagnosis of HPA is critical to distinguish infants with PAH deficiency from those very rare patients with HPA due to BH4 deficiency. Once the initial screening detects HPA in a proband and the diagnostic tests demonstrate PAH or BH4 deficiencies, molecular genetics methods are used to confirm these results (Güttler & Guldberg, 2000). Genetics studies in HPA patients are of utmost importance since not only they can contribute to the therapeutic response prediction, but also for adequate genetic counseling.

## INFORMED CONSENT

Informed was obtained from all patients and their families for being included in the study.

## CONFLICT OF INTEREST

The authors declare that they have no competing interests and that no financial support was obtained for the publication of this manuscript.

## AUTHOR CONTRIBUTIONS

FF, LA, and LV contributed to the concept and designed of research as well as with acquisition, analysis, or interpretation of the data. FF, LA, RN, CS, HF, AM, HR, CC, and SR, contributed to the manuscript preparation. AB, EM, TC, ER, PG, LD, ACF, SS, FS, LR, AG, and PJ, screened the cohorts of patients and evaluated the clinical data. All authors read and approved the final manuscript.

## Supporting information

Table S1

## Data Availability

The data that support the findings of this study are available from the corresponding author upon reasonable request.
