# Supplementary material for: Phenylketonuria in Portugal: Genotype–phenotype correlations using molecular, biochemical, and haplotypic analyses
Source: Mol Genet Genomic Med. 2021 Jan 19;9(3):e1559. doi: 10.1002/mgg3.1559 (PMC8104178; doi:10.1002/mgg3.1559)
Supplement: Supplementary file 1 — Table S1 [file MGG3-9-e1559-s001.docx]

**Supp. Table S1**

Supplementary Data

Table S1 –Genotype characterization of the Portuguese PKU patients, allelic frequency and correlation with the biochemical marker of the disease, increased level of plasmatic phenylalanine at the neonatal screening.

|  | **Allelle1** | **Allele 2** | **Nº patients** | **Phe (mg/dL)** | **Allelic Frequency (%)** |
| --- | --- | --- | --- | --- | --- |
| **Homozygous** | p.(Asp129Tyr) | p.(Asp129Tyr) | 2 | 4.5 | 0.90 |
|  | p.(Ile65Thr) | p.(Ile65Thr) | 3 | 17.7 | 1.35 |
|  | c.1066-11G>A | c.1066-11G>A | 10 | 15.3 | 4.48 |
|  | c.168+5G>A | c.168+5G>A | 1 | 26.6 | 0.45 |
|  | c.441+5G>T | c.441+5G>T | 1 | 31.2 | 0.45 |
|  | p.(Leu249Phe) | p.(Leu249Phe) | 4 | 10.5 | 1.79 |
|  | p.(Phe410Cys) | p.(Phe410Cys) | 1 | - | 0.45 |
|  | p.(Arg176*) | p.(Arg176*) | 2 | 19.9 | 0.90 |
|  | p.(Pro281Leu) | p.(Pro281Leu) | 1 | 21.0 | 0.45 |
|  | p.(Arg158Gln) | p.(Arg158Gln) | 6 | 19.9 | 2.69 |
|  | p.(Arg252Trp) | p.(Arg252Trp) | 3 | 19.0 | 1.35 |
|  | p.(Arg261Gln) | p.(Arg261Gln) | 17 | - | 7.62 |
|  | p.(Val388Met) | p.(Val388Met) | 5 | 12.7 | 2.24 |
|  | p.(Arg68Ser) | p.(Arg68Ser) | 1 | 9.5 | 0.45 |
|  | p.(Arg243*) | p.(Arg243*) | 1 | 17.5 | 0.45 |
|  | p.(Arg270Lys) | p.(Arg270Lys) | 1 | 21.0 | 0.45 |
| **Heterozygous** | p.(Phe39Leu) | c.842+1G>A | 1 | 38.5 | 0.45 |
|  | p.(Arg53His) | p.(Leu348Val) | 1 | 4.3 | 0.45 |
|  | p.(Phe55Leu) | p.(Arg270Lys) | 1 | 4.9 | 0.45 |
|  | p.(Phe55Leufs*6) | p.(Ala403Val) | 1 | 5.3 | 0.45 |
|  | p.(Phe55Leufs*6) | p.(Ala300Ser) | 1 | 2.8 | 0.45 |
|  | p.(Phe55Leufs*6) | c.1066-11G>A | 2 | 29.1 | 0.90 |
|  | p.(Gly352Valfs*12) | p.(Ile421Thr) | 1 | 3.6 | 0.45 |
|  | p.(Gly46Ser) | p.(Arg261Gln) | 1 | 29.6 | 0.45 |
|  | p.(Gly46Ser) | p.(Ile164Val) | 1 | 11.6 | 0.45 |
|  | p.(Ile65Thr) | p.(Arg270Lys) | 1 | 26.2 | 0.45 |
|  | p.(Ile65Thr) | p.(Leu249Phe) | 1 | 12.1 | 0.45 |
|  | p.(Ile65Thr) | p.(Asp129Gly) | 1 | 11.9 | 0.45 |
|  | p.(Ile65Thr) | p.(Arg176Leu) | 2 | - | 0.90 |
|  | p.(Ile65Thr) | p.(Asp145Val) | 1 | 3.7 | 0.45 |
|  | p.(Ile65Thr) | p.(Ile164Val) | 1 | 5.2 | 0.45 |
|  | p.(Ile65Thr) | p.(Arg261Gln) | 5 | 13.4 | 2.24 |
|  | p.(Ile65Thr) | c.1066-11G>A | 3 | 21.3 | 1.35 |
|  | p.(Ile65Thr) | p.(Ala300Ser) | 1 | 6.9 | 0.45 |
|  | p.(Ile65Thr) | p.(Pro281Leu) | 2 | 19.5 | 0.90 |
|  | p.(Ile65Thr) | p.(Arg252Trp) | 2 | - | 0.90 |
|  | p.(Ile65Thr) | p.(Asp145Val) | 1 | 4.7 | 0.45 |
|  | p.(Ile65Thr) | p.(Arg270Lys) | 1 | 8.7 | 0.45 |
|  | p.(Ile65Thr) | p.(Arg243Gln) | 1 | 2.41 | 0.45 |
|  | p.(Ile65Thr) | p.(Pro281Leu) | 1 | 13.8 | 0.45 |
|  | p.(Ile65Thr) | p.(Leu367Gln) | 1 | 10 | 0.45 |
|  | p.(Ile65Thr) | c.168+5G>A | 1 | 8.5 | 0.45 |
|  | p.(Ile65Thr) | c.1199+5G>A | 1 | 21 | 0.45 |
|  | p.(Arg68Ser) | p.(Arg270Lys) | 1 | 6.9 | 0.45 |
|  | p.(Arg68Ser) | p.(Arg158Gln) | 1 | 12 | 0.45 |
|  | p.(Arg68Ser) | p.(Arg241His) | 1 | 5.6 | 0.45 |
|  | p.(Arg68Ser) | c.1315+1G>A | 1 | 6.7 | 0.45 |
|  | p.(Arg68Ser) | IVS10-11G>A | 1 | 14 | 0.45 |
|  | P.(Asp129Tyr) | p.(Leu348Val) | 1 | 7 | 0.45 |
|  | p.(Arg158Gln) | p.(Pro281Leu) | 1 | 21 | 0.45 |
|  | p.(Arg158Gln) | p.(Leu249Phe) | 1 | 11 | 0.45 |
|  | p.(Arg158Gln) | p.(Arg252Trp) | 1 | 8.6 | 0.45 |
|  | p.(Arg158Gln) | p.(Arg297Cys) | 1 | 3 | 0.45 |
|  | p.(Arg158Gln) | p.(Ala403Val) | 1 | 2.9 | 0.45 |
|  | p.(Arg158Gln) | p.(Pro281Leu) | 2 | 19.4 | 0.90 |
|  | p.(Arg158Gln) | p.(Arg261Gln) | 1 | 24.5 | 0.45 |
|  | p.(Arg158Gln) | c.1315+1G>A | 2 | 31.0 | 0.90 |
|  | p.(Arg158Gln) | c.168+5G>C | 1 | 18.1 | 0.45 |
|  | p.(Arg176Leu) | p.(Arg261Gln) | 2 | 4.3 | 0.90 |
|  | p.(Arg176Leu) | p.(Arg252Trp) | 3 | 5.7 | 1.35 |
|  | p.(Arg176Leu) | p.(Leu249Phe) | 1 | 2.5 | 0.45 |
|  | p.(Arg176Leu) | P.(Val388Met) | 1 | 5 | 0.45 |
|  | p.(Arg176Leu) | p.(Arg243Gln) | 1 | 5.8 | 0.45 |
|  | p.(Arg176Leu) | p.(Arg270Lys) | 1 | 5.4 | 0.45 |
|  | p.(Arg176Leu) | p.(Pro281Leu) | 1 | 4.7 | 0.45 |
|  | p.(Arg176Leu) | c.1066-11G>A | 5 | 8.4 | 2.24 |
|  | p.(Arg176Leu) | c.168+5G>A | 2 | 6.7 | 0.90 |
|  | p.(Arg176*) | p.(Arg261Gln) | 1 | 10.6 | 0.45 |
|  | p.(Arg176*) | p.(Ala309Val) | 1 | 16.7 | 0.45 |
|  | p.(Arg176*) | P.(Val388Met) | 2 | 20.8 | 0.90 |
|  | p.(Arg176*) | p.(Ala403Val) | 1 | 5.7 | 0.45 |
|  | p.(Arg176*) | c.1066-11G>A | 2 | 18.6 | 0.90 |
|  | p.(Glu178Gly) | p.(Ala309Asp) | 1 | 6.45 | 0.45 |
|  | p.(Glu182Lys) | p.(Leu348Val) | 1 | 10 | 0.45 |
|  | p.(Val230Ile) | p.(Leu348Val) | 1 | 4.5 | 0.45 |
|  | p.(Arg243Gln) | p.(Asp145Asn) | 1 | 7.5 | 0.45 |
|  | p.(Arg243Gln) | p.(Pro281Leu) | 1 | 30 | 0.45 |
|  | p.(Leu249Phe) | p.(Phe410Cys) | 1 | 6.6 | 0.45 |
|  | p.(Leu249Phe) | p.(Ala300Ser) | 3 | 4.0 | 1.35 |
|  | p.(Leu249Phe) | p.(Leu348Val) | 2 | 13.4 | 0.90 |
|  | p.(Leu249Phe) | p.(Arg261*) | 1 | 19.4 | 0.45 |
|  | p.(Leu249Phe) | p.(Ala309Val) | 1 | 12.5 | 0.45 |
|  | p.(Leu249Phe) | p.(Pro281Leu) | 2 | 9.3 | 0.90 |
|  | p.(Leu249Phe) | c.1066-11G>A | 1 | 20 | 0.45 |
|  | p.(Arg252Trp) | p.(Arg270Lys) | 1 | 13.2 | 0.45 |
|  | p.(Arg252Trp) | p.(Ala300Ser) | 1 | 4.8 | 0.45 |
|  | p.(Arg252Trp) | c.1066-11G>A | 2 | 22.5 | 0.90 |
|  | p.(Arg261Gln) | p.(Asp145Asn) | 2 | 7.2 | 0.90 |
|  | p.(Arg261Gln) | p.(Ala403Val) | 1 | 6.2 | 0.45 |
|  | p.(Arg261Gln) | p.(Ser87Arg) | 2 | 5.2 | 0.90 |
|  | p.(Arg261Gln) | p.(Glu390Gly) | 1 | 6.8 | 0.45 |
|  | p.(Arg261Gln) | p.(Pro281Leu) | 1 | 12 | 0.45 |
|  | p.(Arg261Gln) | p.(Val388Met) | 2 | 22.7 | 0.90 |
|  | p.(Arg261Gln) | p.(Leu308Phe) | 1 | 13.9 | 0.45 |
|  | p.(Arg261Gln) | p.(Asp129Gly) | 1 | 7.1 | 0.45 |
|  | p.(Arg261Gln) | p.(Leu348Val) | 1 | 14.5 | 0.45 |
|  | p.(Arg261Gln) | p.(Leu48Ser) | 1 | 5.2 | 0.45 |
|  | p.(Arg261Gln) | p.(Asp145Val) | 1 | 6.5 | 0.45 |
|  | p.(Arg261Gln) | c.1066-11G>A | 2 | 17.5 | 0.90 |
|  | p.(Arg261Gln) | p.(Arg243Gln) | 1 | 28.7 | 0.45 |
|  | p.(Arg261Gln) | c.168+5G>A | 1 | 11.2 | 0.45 |
|  | p.(Arg270Lys) | p.(Ala403Val) | 1 | 2.7 | 0.45 |
|  | p.(Arg270Lys) | p.(Glu390Gly) | 1 | 11 | 0.45 |
|  | p.(Arg270Lys) | c.1066-11G>A | 1 | 11.1 | 0.45 |
|  | p.(Val388Met) | p.(Arg252Trp) | 2 | 23.7 | 0.90 |
|  | p.(Val388Met) | p.(Arg297Cys) | 2 | 4.1 | 0.90 |
|  | p.(Val388Met) | p.(Ala403Val) | 2 | 7.9 | 0.90 |
|  | p.(Val388Met) | p.(Tyr414Cys) | 1 | 6.9 | 0.45 |
|  | p.(Val388Met) | p.(Glu390Gly) | 3 | 3.6 | 1.35 |
|  | p.(Val388Met) | c.1066-11G>A | 5 | 12.0 | 2.24 |
|  | p.(Val388Met) | c.168+5G>A | 2 | 8.5 | 0.90 |
|  | p.(Val388Met) | p.(Arg408Trp) | 1 | - | 0.45 |
|  | p.(Val388Met) | p.(Tyr414Cys) | 2 | 13.5 | 0.90 |
|  | p.(Val388Met) | p.(Pro281Leu) | 1 | 9.5 | 0.45 |
|  | p.(Ala322Gly) | p.(Val388Met) | 1 | 4.6 | 0.45 |
|  | p.(Ala359Phe) | p.(Val388Met) | 1 | 9 | 0.45 |
|  | p.(Tyr414Cys) | p.(Arg408Trp) | 1 | 16 | 0.45 |
|  | c.1066-11G>A | p.(Arg297Cys) | 2 | 5.2 | 0.90 |
|  | c.1066-11G>A | p.(Arg297His) | 1 | 5.5 | 0.45 |
|  | c.1066-11G>A | p.(Leu348Val) | 4 | 14.2 | 1.79 |
|  | c.1066-11G>A | p.(Pro281Leu) | 1 | 25.6 | 0.45 |
|  | c.1066-11G>A | p.(Arg243Gln) | 1 | 21.8 | 0.45 |
|  | c.1066-11G>A | p.(Asp129Tyr) | 1 | 10.6 | 0.45 |
|  | c.1066-11G>A | p.Ala403Val | 3 | 3.7 | 1.35 |
|  | c.1066-11G>A | c.1315+1G>A | 1 | 27 | 0.45 |
|  | c.1066-11G>A | p.(Ala300Ser) | 1 | 2.8 | 0.45 |
|  | c.1066-11G>A | p.(Ala313Val) | 1 | 4 | 0.45 |
|  | c.1066-11G>A | p.(Tyr414Cys) | 1 | 13.9 | 0.45 |
|  | c.168+5 G>C | p.(Ala300Ser) | 1 | 4.6 | 0.45 |

(GenBank: NM_000277.3; ENSG00000171759; ENST00000553106.6)
